# Direct therapeutic effect of sulfadoxine-pyrimethamine on nutritional deficiency-induced enteric dysfunction in a human Intestine Chip

**DOI:** 10.1016/j.ebiom.2023.104921

**Published:** 2023-12-14

**Authors:** Seongmin Kim, Arash Naziripour, Pranav Prabhala, Viktor Horváth, Abidemi Junaid, David T. Breault, Girija Goyal, Donald E. Ingber

**Affiliations:** a Wyss Institute for Biologically Inspired Engineering, Harvard University, Boston, MA 02115, USA; b Department of Pediatrics, Harvard Medical School, Boston, MA 02115, USA; c Department of Endocrinology, Boston Children’s Hospital, Boston, MA 02115, USA; d Harvard Stem Cell Institute, Cambridge, MA 02138, USA; e Vascular Biology Program, Boston Children’s Hospital and Department of Pathology, Harvard Medical School, Boston, MA 02115, USA; f Harvard John A. Paulson School of Engineering and Applied Sciences, Cambridge, MA 02139, USA

**Keywords:** Sulfadoxine-pyrimethamine, Malnutrition, Enteric dysfunction, Organ-on-a-chip, Birth outcomes

## Abstract

**Background:**

Sulfadoxine-pyrimethamine (SP) antimalarial therapy has been suggested to potentially increase the birth weight of infants in pregnant women in sub-Saharan Africa, independently of malarial infection. Here, we utilized female intestinal organoid-derived cells cultured within microfluidic Organ Chips to investigate whether SP could directly impact intestinal function and thereby improve the absorption of essential fats and nutrients crucial for fetal growth.

**Methods:**

Using a human organ-on-a-chip model, we replicated the adult female intestine with patient organoid-derived duodenal epithelial cells interfaced with human intestinal endothelial cells. Nutrient-deficient (ND) medium was perfused to simulate malnutrition, resulting in the appearance of enteric dysfunction indicators such as villus blunting, reduced mucus production, impaired nutrient absorption, and increased inflammatory cytokine secretion. SP was administered to these chips in the presence or absence of human peripheral blood mononuclear cells (PBMCs).

**Findings:**

Our findings revealed that SP treatment effectively reversed multiple intestinal absorptive abnormalities observed in malnourished female Intestine Chips, as validated by transcriptomic and proteomic analyses. SP also reduced the production of inflammatory cytokines and suppressed the recruitment of PBMCs in ND chips.

**Interpretation:**

Our results indicate that SP could potentially increase birth weights by preventing enteric dysfunction and suppressing intestinal inflammation. This underscores the potential of SP as a targeted intervention to improve maternal absorption, subsequently contributing to healthier fetal growth. While SP treatment shows promise in addressing malabsorption issues that can influence infant birth weight, we did not model pregnancy in our chips, and thus its usefulness for treatment of malnourished pregnant women requires further investigation through clinical trials.

**Funding:**

The 10.13039/100000865Bill and Melinda Gates Foundation, and the 10.13039/100016528Wyss Institute for Biologically Inspired Engineering at 10.13039/100007229Harvard University, and the 10.13039/100020141HDDC Organoid Core of the P30 DK034854.

**Research in context**

**Evidence before this study**

A previous clinical study found that prophylactic treatment of pregnant mothers with sulfadoxine-pyrimethamine (SP) reduced the incidence of low birth weight infants even in the absence of malarial infection and when the organism exhibited SP resistance. These findings suggest that SP might have a direct effect on intestinal absorption in the adult female intestine and thereby positively impact newborn birth weight.

**Added value of this study**

In this study, we assessed the direct effects of SP on intestinal function using human organ-on-a-chip (Organ Chip) technology. We created adult female Intestine Chips with patient-derived intestinal epithelium, interfaced with intestinal microvascular endothelium in the presence or absence of immune cells, and modeled malnutrition by culturing them in nutrient-deficient (ND) medium lacking niacinamide and tryptophan. Healthy and ND Intestine Chips were then used to evaluate the impact of SP on intestinal absorption and pathophysiology. SP treatment was found to have direct beneficial effects on human intestinal structure, function, and inflammation state, potentially explaining improved newborn birth weight in pregnant women receiving this therapy. While SP treatment shows promise in addressing malabsorption issues that can influence infant birth weight, further in-depth clinical studies are needed to determine its efficacy in treating malnourished pregnant women.

**Implications of all the available evidence**

This study evaluates intestinal function under malnutrition conditions and the impacts of SP treatment in vitro. Our findings indicate that SP has multiple direct effects on human intestinal structure and function, potentially explaining the reduced incidence of low birth weight newborns after anti-malaria treatment with SP in pregnant women. We hope these results will encourage further formal clinical studies to validate the efficacy and safety of SP as a potential direct intervention for this global health issue. Additionally, the human Intestine Chip may offer insights into other questions related to intestinal physiology and pathophysiology relevant to the global health community.

## Introduction

Malnutrition during pregnancy, particularly a lack of vital nutrients like iron, fat, calcium, and zinc, can lead to adverse birth outcomes such as low birth weight and developmental delays in children.^1^^,^^2^ Low birth weight is also an important indicator of infant mortality, which can be caused by preterm birth, small size for gestational age, and malaria.^2^ To combat malaria infection and reduce the risk of adverse birth outcomes, the World Health Organization (WHO) recommends intermittent preventive treatment with combined sulfadoxine and pyrimethamine (SP) during pregnancy.^3^ Interestingly, a recent study found that maternal SP treatment can reduce the incidence of low birth weight infants even in the absence of malarial infection and when the organism is resistant to SP treatment.^4^ The physiological relevance of this observation remains unclear as there is limited understanding of the effects of SP treatment on maternal health and birth outcomes.

In this study, we aimed to investigate the effects of SP treatment on nutrient absorption in the adult female small intestine and its impact on intestinal function using functional, transcriptomic, and metabolic assays. To achieve this, we employed a microfluidic human organ-on-a-chip model of the Intestine (Intestine Chip) that is lined by human intestinal epithelium derived from organoids isolated from the duodenum of young adult females interfaced with primary human intestinal microvascular endothelium. This model allowed us to directly assess the potential therapeutic effects of SP on the intestine. To simulate malnutrition in the adult female intestine, we exposed the Intestine Chip to nutrient-deficient (ND) medium lacking niacinamide and tryptophan. We have previously demonstrated that human Intestine Chips cultured under these ND conditions exhibited characteristic signs of environmental enteric dysfunction (EED), such as villus blunting, decreased mucus production, and reduced absorption of fatty acids and peptides, as well as altered transcriptomic profiles, consistent with previous clinical findings in EED patients.^5^ Given the finding that SP seems to increase birth weight in maternal patients who are resistant to the anti-malarial effects of this drug,^4^ we administered SP through the apical epithelium-lined channel of the Intestine Chip to mimic oral administration in patients and observed its effects under both healthy and ND conditions. Recognizing the complex interplay between intestinal epithelial cells and the mucosal immune system,^6^^,^^7^ we also conducted experiments using human peripheral blood mononuclear cells (PBMCs) introduced into the basal endothelium-lined channel of the Intestine Chips. Our results demonstrate that SP has direct protective effects on the malnourished adult female intestine.

## Methods

### Preparation of Intestine Chips and SP treatment

In this study, we used three different duodenal organoids samples, each from a different aged young woman (14, 18, and 21 years old). Our methods for culturing patient-derived epithelial intestinal organoids, enzymatically releasing the cells, and then culturing them within one channel of a two-channel microfluidic organ chip device (Emulate Inc.) separated by a porous membrane with 7 μm pore diameter from primary human microvascular endothelial cells in the second channel, have been described previously.^5^^,^^8^ The upper epithelial channel was perfused with complete medium or ND medium without niacinamide and tryptophan at 60 μL/h to mimic the EED state, while the lower channel was perfused with expansion culture medium. Sulfadoxine (S; 200 mg, USP) and pyrimethamine (P; 200 mg, USP) were prepared in 0.5 mL DMSO (ThermoFisher), and mixed with differentiation medium (DM) at a ratio of 20:1 (133 μg S + 6.7 μg P)/mL which simulates the acute duodenal exposure at the WHO recommended dose (500 mg S + 25 mg P/tablet) of the SP formulation (192 μg S + 9.6 μg P)/chip for 1 day; equivalent to 1 SP tablet in 250 mL gastric volume considering the gastric volume of a healthy adult (250–300 mL); these drugs were then perfused through the epithelial channel for 3 days.

### Ethics

Intestinal organoids were generated from biopsy samples from healthy regions of the duodenum in patients being evaluated for potential other intestinal problems, as described,^5^^,^^9^ and the written informed consent was obtained from all participants. All methods were performed according to the approval of the Institutional Review Board of Boston Children’s Hospital (Protocol number IRB-P00000529).

### Morphological analysis

For the villus height measurements, each datapoint represents the average of 5 measurement points randomly selected from 2 to 3 cross-sectional images of each chip, and this analysis was performed on at least 3 chips per experimental condition. For differential interference contrast (DIC) and immunofluorescence microscopic imaging, tissues within either whole or Vibratome (Leica VT1000S) sectioned chips were fixed with 4% paraformaldehyde (PFA), permeabilized, and blocked with 0.1% Triton X-100 solution and 10% donkey serum in PBS. F-actin (Invitrogen, O7466) and villin (Invitrogen, PA5-29078) immunostaining studies were carried out to visualize microvilli. Barrier integrity was assessed using antibodies directed against ZO-1 (Invitrogen, 33-9100), VE-Cadherin (ThermoFisher, ab33168), and species-specific secondary antibodies (Invitrogen, A31570 and Invitrogen, A31571); nuclei were stained with Hoechst 33342 (Invitrogen, H3570). Pseudo hematoxylin and eosin (H&E) staining was carried out on 40–60 μm chip sections, as described.^10^ Imaging was carried out using a laser scanning confocal microscope (Leica SP5 X MP DMI-6000), and IMARIS (Ver. 9.9.1, Oxford Instruments) and ImageJ software were used for processing and analysis of 3D high-resolution horizontal or vertical cross-sectional images.

### Intestinal barrier permeability

We assessed the paracellular permeability of the intestinal barrier within Intestine Chips as previously described using the small fluorescent biomarker, Cascade Blue (596 Da; ThermoFisher, C687).^11^ Briefly, the apical-to-basolateral flux of the paracellular marker was calculated using the following formula: *P*_*app*_ = (*V*_*r*_ × *C*_*r*_)/(*t* × *A* × *Cd out*), where *V*_*r*_ is the volume of the receiving channel outflow, *C*_*r*_ is the concentration of tracer in the receiving channel, *t* is time (sec), *A* is the total area of diffusion (cm^2^), and *Cd out* is the concentration of tracer in the dosing channel outflow (mg/mL).

### Mucus accumulation

Intestinal mucus was visualized on-chip using wheat germ agglutinin (WGA)-Alexa 488 (ThermoFisher).^5^ Briefly, 25 μg/mL of WGA in Hanks’ Balanced Salt Solution (HBSS, Gibco) was flowed at 200 μL/h through the apical channel for 30 min and then washed with continuous flow of HBSS at the same flow rate for 30 min. The entire channel was visualized with a fluorescence microscope (Excitation/Emission = 488/523 nm, Zeiss Axio Observer Z1). The mucus layer was set as the distance between the apical cell surface and the upper limit of the WGA-labeled material; the thickness of the mucus layer was measured by ImageJ. For MUC2 visualization, immunofluorescence microscopic imaging was carried out on 150–200 μm chip sections using antibodies against MUC2 (Santa Cruz, sc-15334) and a secondary antibody (ThermoFisher, A31573).^12^^,^^13^

### Fatty acid uptake

Fatty acid (FA) absorption was measured as described.^5^ Briefly, the media in the apical and basal channels of the Intestine Chips were switched to HBSS for 1 h. A fluorescently labeled dodecanoic acid containing a quencher (10 μL quencher + 5 μL FA + 485 μL assay buffer + 500 μL HBSS) was then added to the apical channel according to the manufacturer’s instructions (BioVision, K408). Fluorescence microscopic imaging of the epithelial channel was then carried out (Excitation/Emission = 488/523 nm, Zeiss Axio Observer Z1 and Echo Revolve microscope) and FA uptake was calculated by determining fluorescence intensity levels using ImageJ. For visualization of intracellular uptake of fluorescently labeled FA, cells within vertical 150–200 μm thick cryosections of the chips were imaged with a laser scanning confocal microscope (Zeiss, LSM 980).

### Transcriptomic analysis

RNA sequencing analysis of epithelial cells removed from the chips by exposure to Accumax™ (STEMCELL Technologies) was performed by AZENTA Life Sciences (NJ, USA). Briefly, RNA was isolated using RNeasy Plus Micro Kit (Qiagen) and the samples were evaluated for RNA quality. Libraries were sequenced using the Illumina HiSeq4000 platform, and the resulting150 bp long pair-ended reads were trimmed using Trimmomatic (v.0.36) mapped to GRCh38 reference genome using STAR (v.2.5.2); gene hit counts to ENSEMBL Release 105 transcriptome annotation were generated using featureCounts of Subread package (v.1.5.2) for downstream analysis. Differential gene expression analysis was performed using DESeq2 (v.1.26.0) with the Benjamini and Hochberg method for adjusting *p*-values for multiple hypothesis testing. Genes with adjusted *p*-values <0.05 and absolute log_2_(fold change) ≥1 were considered differentially expressed. Relevant biological pathways were identified in the MSigDB Hallmark 2020 database using gene set enrichment analysis (GSEA).^14^ For TaqMan qPCR assay, total RNA was extracted from epithelial cells removed from the chips using RNeasy Micro Kit (Qiagen) and complementary DNA was prepared using Omniscript RT Kit (Qiagen). The qPCR assay was performed on the QuantStudio™ 7 Flex (Applied biosystems) with TaqMan™ Fast Advanced Master Mix (Applied biosystems) for DMT1 (ThermoFisher, Hs00167206_m1) and FPN (ThermoFisher, Hs00205888_m1). Expression levels of target genes were normalized to beta actin (ACTB).

### Inflammatory cytokine production

Changes in cytokine levels were measured in apical and basal channel effluents from our chips.^11^ Ten inflammatory cytokines were selected and a Luminex assay was conducted according to the manufacturer’s protocol (Invitrogen). Analyte concentrations were measured using a Luminex FLEXMAP 3D instrument with xPONENT® software. For apoptosis analysis, cleaver Caspase-3 (Cell Signaling Technology, 9661S) was stained with a secondary antibody (ThermoFisher, A31572) and imaged with a laser scanning confocal microscope (Leica); the fluorescence intensity was measured using ImageJ.

### Immune cell recruitment

We quantified the effects of SP on the recruitment of immune cells through the endothelium and to the epithelium by perfusing PBMCs through the lower endothelium-lined channel, as previously described.^11^ Briefly, de-identified human patient-derived apheresis collars were obtained from the Crimson Biomaterials Collection Core Facility under approval obtained from the Institutional Review Board at Harvard University (#22470). Cells were stained with CellTracker Green™ CMFDA (1:1000 v/v in PBS, ThermoFisher, #C7025) for 10 min at 37°C in a water bath and stained PBMCs were then seeded into the basal channel (endothelium) of the Intestine Chips at 5 × 10^7^ cells/mL in medium. PBMCs were allowed to adhere to the endothelium by inverting the chips for 3 h under a static condition, were gently washed with complete medium to remove non-adherent or suspended cells, and were then maintained at continuous flow (60 μL/h). The number of PBMCs that adhered and migrated from the basal vascular channel to the apical epithelium-lined channel was quantified by immunofluorescence microscopic imaging. The composition of these PBMCs recruited to the epithelium was determined using Flow Cytometry (CytoFlex LX); cell digests were stained with Viakrome 808 Live/Dead stain (Beckman Coulter, C36628), CD19 BV421 (BioLegend, 302234), CD3 APC-H7(BD Biosciences, 560176), and CD14 BV395 (BD Biosciences, 563561), and then fixed with 200 μL of Cytofix (BD Biosciences, 554655). To calculate the absolute number of cells per sample, 5025 counting beads were added to each sample. Results were analyzed using FlowJo V10 software (FlowJo, LLC).

### Statistical analysis

Between 3 and 6 Intestine Chips were used in each study and a one-way ANOVA was performed to determine statistical significance, as indicated in the figure legends. *p* < 0.05 were considered significant; error bars indicate mean ± standard deviation (s.d.).

### Role of funders

This study was funded by the Bill and Melinda Gates Foundation, Wyss Institute for Biologically Inspired Engineering at Harvard University, and the HDDC Organoid Core of the P30 DK034854. The funding sources did not play a role in the study design, data collection, data analyses, interpretation, or writing of the manuscript.

## Results

### SP mitigates the effects of nutritional deficiency in the human Intestine Chip

**Fig. 1:**
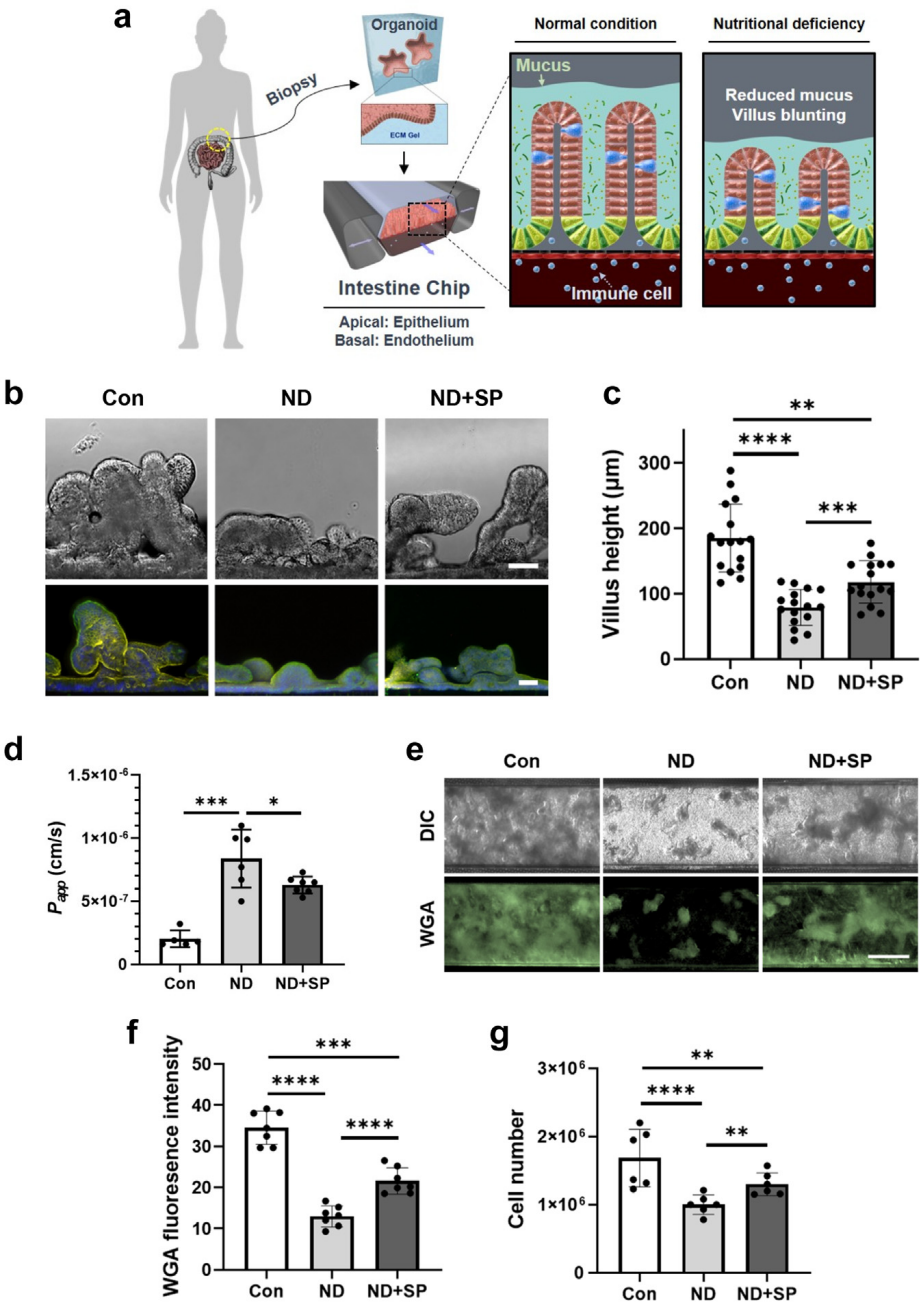
**Effect of nutritional deficiency and SP treatment on adult female Intestine Chip.** (a) A schematic cross-sectional view of the duodenal organoid-derived Intestine Chip showing the apical (epithelium) and basal (endothelium) microchannels. The sulfadoxine-pyrimethamine (SP) drug combination was applied to the apical channel and the human peripheral blood mononuclear cells (PBMCs) were applied to the basal channel. (b) DIC and immunofluorescence imaging of cross-sectioned Intestine Chips showing villus-like structures, green: phalloidin, yellow: ZO-1, blue: Hoechst 33342. Scale bar = 50 μm. (c) Differences in villus-like structure height between normal (Con), nutrient-deficient (ND), and SP-treated ND (ND + SP) Intestine Chips. ∗∗*p* < 0.01, ∗∗∗*p* < 0.001, ∗∗∗∗*p* < 0.0001 by a one-way ANOVA. (d) Apparent permeability (*P*_*app*_) after SP treatment. ∗*p* < 0.05, ∗∗∗*p* < 0.001 by a one-way ANOVA. (e) Immunofluorescence imaging of Intestine Chips stained with wheat germ agglutinin (WGA) with Alexa-488 conjugated lectin showing mucus production. Scale bar = 100 μm. (f) Differences in fluorescence intensity of WGA. ∗∗∗*p* < 0.001, ∗∗∗∗*p* < 0.0001 by a one-way ANOVA. (g) Comparison of cell number between Con, ND, and ND + SP Intestine Chips. ∗∗*p* < 0.01, ∗∗∗∗*p* < 0.0001 by a one-way ANOVA.

In this study, we set out to address the adverse effects of ND conditions using an Intestine Chip model that faithfully replicates the structure and functionality of the human intestine (Fig. 1a).^8^ Primary duodenal epithelial cells isolated from patient-derived organoids were cultured on the upper extracellular matrix-coated surface of the porous membrane that separates two parallel microchannels within the chips and primary human intestinal microvascular endothelium was grown on the lower surface of the same membrane. The culture medium was continuously perfused through both channels while peristalsis-like deformations were produced by the application of cyclic suction to side chambers within the flexible polymer chip. Recreation of the intestinal microenvironment in this manner leads to the spontaneous formation of intestinal villi-like structures (Fig. 1b and c, and Supplementary Figure S1a). The chip also maintained an intact intestinal barrier (Fig. 1d) and actively produced mucus (Fig. 1e and f).

To simulate malnutrition conditions, we perfused the chips with ND medium which lacked the essential nutrients niacinamide and tryptophan. This resulted in villus blunting (Fig. 1b and c, and Supplementary Figure S1a), compromised barrier function (Fig. 1d), and reduced mucus production (Fig. 1e and f), as previously described.^5^ Consistent with reduced barrier function, histological and immunofluorescence microscopic analysis revealed that exposure to ND resulted in epithelial and cytoskeletal damage (Supplementary Figure S1a), as well as partial disruption of VE cadherin-containing endothelial cell-cell contacts (Supplementary Figure S1b).

Considering the WHO recommendation of a high single dose (500 mg S + 25 mg P) of the SP drug combination during pregnancy, we perfused the epithelial channel of the Intestine Chip with the calculated acute duodenal dose ([133 μg S + 6.7 μg P]/mL) of this formulation as well as lower doses (1/50th [2.66 μg S + 0.13 μg P]/mL and 1/10th [13.3 S + 0.67 μg P]/mL) in culture medium and assessed its effects on morphology, barrier function, and cytokine production. We did not observe any significant changes in barrier permeability, epithelial morphology, or inflammatory cytokine production in control Intestine Chips cultured with any of the SP doses when tested in culture medium for three days (Supplementary Figure S2a–c). In contrast, SP treatment resulted in a significant reversal of the ND phenotype in the malnourished Intestine Chip. This was evidenced by increased villus height (Fig. 1b and c, and Supplementary Figure S1a), improved barrier function (Fig. 1d), and enhanced mucus production (Fig. 1e and f) with increased accumulation of MUC2 (Supplementary Figure S4b). In addition, when we stained the chips with fluorescently-labeled Wheat Germ Agglutinin (WGA) to visualize mucins, we observed a layer of brightly staining mucus covering the surface of the intestinal epithelium, with a notable reduction in mucus thickness observed in ND chips compared to both healthy controls and ND + SP chips (Supplementary Figure S3a and b). We also observed significant loss and morphological changes in the intestinal microvilli in ND chips, whereas SP treatment appeared to ameliorate this microvillar damage (Supplementary Figure S4a and b). Consistent with these findings, key genes associated with microvilli (MYO1A, CDHR2, CDHR5, MYO7B, VIL1) and mucins (MUC2, MUC13, MUC3A, MUC17) were downregulated in ND chips compared to healthy controls whereas SP treatment appeared to partially reverse this effect (Supplementary Figure S4c and d). We also noted a reduction in the total number of epithelial cells in the ND condition, which also was significantly reversed in the presence of SP (Fig. 1g).

### SP increases absorption in nutrient-deficient Intestine Chips

**Fig. 2:**
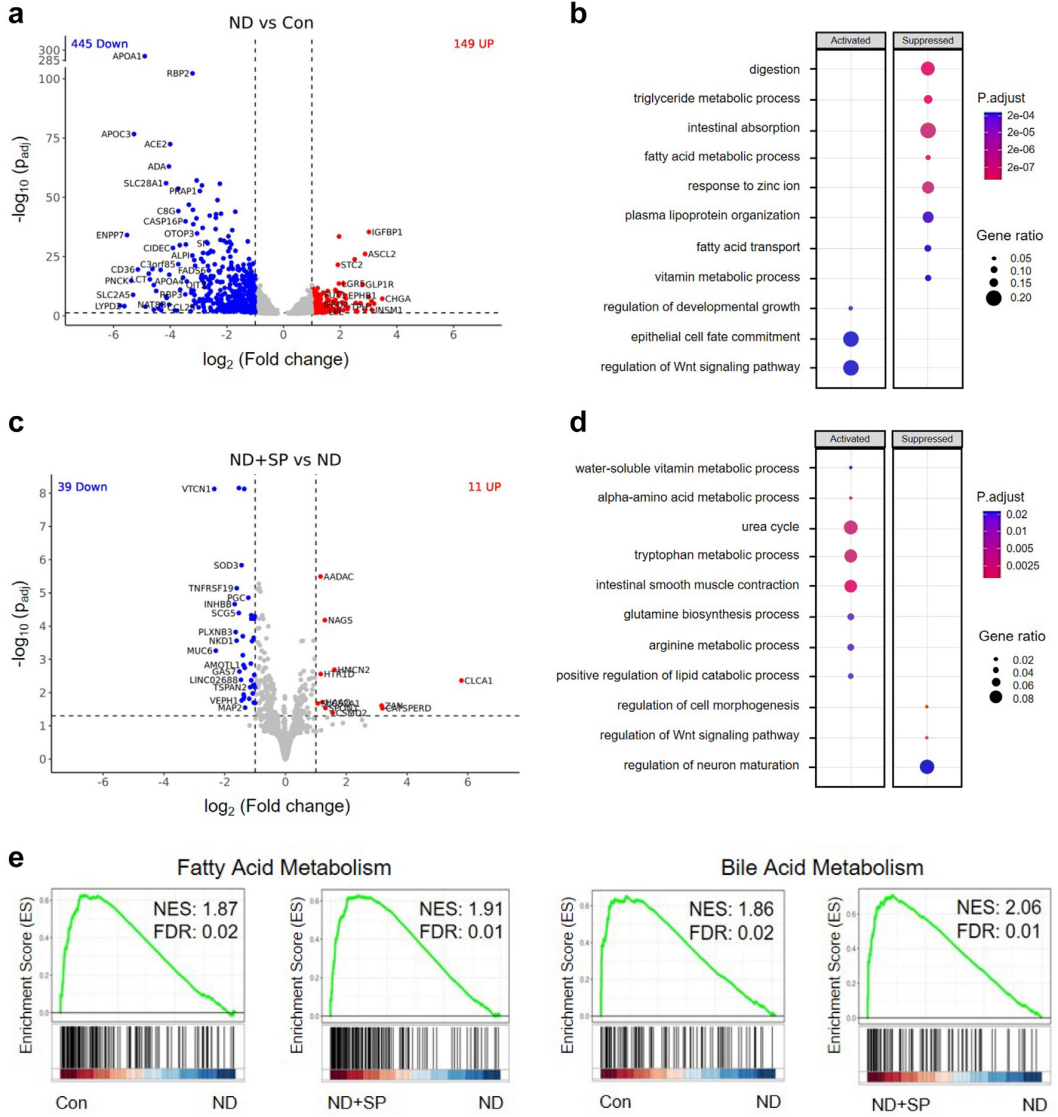
**Reversal of impaired absorption in nutrient-deficient Intestine Chips by SP treatment.** (a) Volcano plot of DEGs from nutrient-deficient (ND) chips compared to control (Con) chips. (b) Dot plot showing the biological processes activated or suppressed by ND vs. Con conditions from (a). (c) Volcano plot of DEGs found in epithelial cells from SP-treated ND chips (ND + SP) compared to those from ND chips. (d) Dot plot showing the biological processes activated or suppressed by ND + SP vs. ND condition from (c). (e) Gene Set Enrichment Analysis (GSEA) plots showing the significant enrichment of gene sets in epithelial cells from Con vs. ND and from ND + SP vs. ND conditions, respectively. MSigDB Hallmark 2020 was used for GSEA plot.

Nutritional components including fatty acids and vitamins help to maintain healthy gut homeostasis and prevent intestinal inflammation, and the absorption of fatty acids, zinc, and triglyceride in pregnant women is crucial for normal fetal tissue development and birth outcomes.^15^^,^^16^ When RNA-seq analysis of epithelial cells obtained from Healthy Intestine Chips, ND chips, and ND chips with SP treatment was carried out, we detected 594 genes (adjusted *p* < 0.05 and log_2_(fold change) ≥ 1.0; 149 upregulated, 445 downregulated) that were differentially regulated in the ND condition compared to healthy controls. Intestinal epithelial cells exposed to ND medium exhibited lower expression of lipoproteins (APOA1, APOC3), solute carrier (SLC) transporters (SLC2A5, SLC6A13, SLC28A1), and mucins (MUC2) compared to healthy chip controls (Fig. 2a). Functional enrichment analysis demonstrated that nutritional deficiency suppressed pathways related to digestion, the triglyceride metabolic process, intestinal absorption, the fatty acid metabolic process, the response to zinc ion, and the vitamin metabolic process, but activated epithelial cell fate commitment and regulation of the Wnt signaling pathway compared with healthy chip controls (Fig. 2b).

**Fig. 3:**
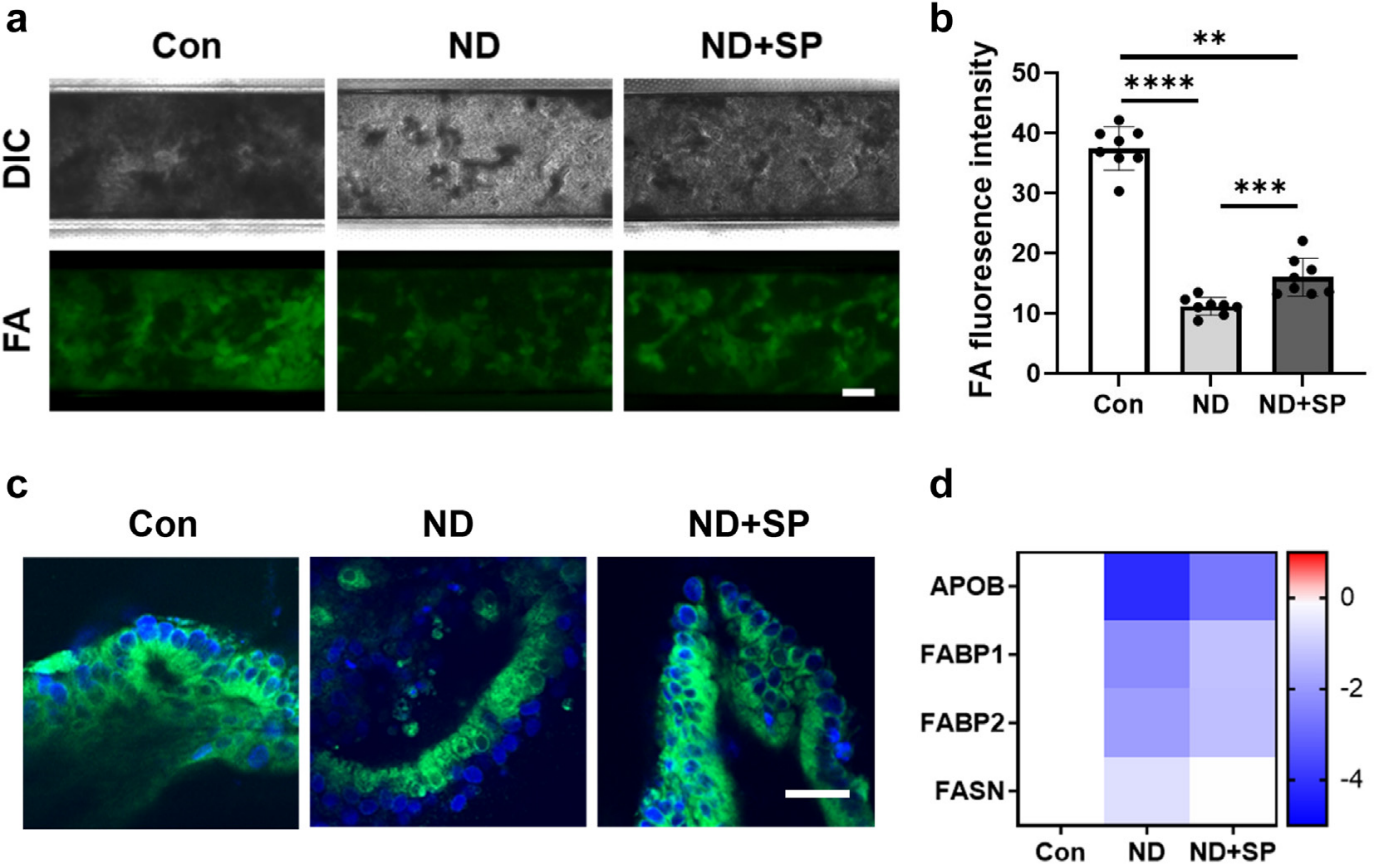
**Impaired fatty acid absorption.** (a) Fatty acid (FA) absorption assay in the Intestine Chips by fluorescently-labeled (Alexa 488) dodecanoic fatty acid uptake at the 60 min time point. Scale bar = 50 μm. (b) Differences in fluorescence intensity of up-taken FA. ∗∗*p* < 0.01, ∗∗∗*p* < 0.001, ∗∗∗∗*p* < 0.0001 by a one-way ANOVA. (c) Intracellular imaging for FA uptake in the Intestine Chips. Scale bar = 50 μm. (d) Heatmap showing differential expression of key genes associated with FA absorption. The color-coded scale represents the log_2_ fold change in expression.

In contrast, when we compared transcriptomic expression profiles from SP-treated ND chips to untreated ND chips, we detected differential expression of 50 genes (adjusted *p* < 0.05 and log_2_(fold change) ≥ 1.0; 11 upregulated, 39 downregulated). The cells from ND + SP chips exhibited higher expression of various genes related to key enzymes in the small intestine (AADAC, NAGS, HMCN2), intestinal mucus homeostasis (CLCA1), a transporter for water-soluble vitamins (SLC52A1), a mediator for iron ion and oxygen binding (HAAO), those involved in cell recognition and behaviors (CATSPERD, ZAN, HTR1D), and protein processing (SPON1) during pregnancy compared to the untreated ND condition (Fig. 2c). Functional enrichment analysis indicated that cells in SP-treated ND chips reactivated various metabolic processes (water-soluble vitamin, alpha-amino acid, tryptophan, arginine, and lipid), and biological pathways (urea cycle, intestinal smooth muscle contraction, and glutamine biosynthesis), but suppressed regulation of the Wnt signaling pathway, cell morphogenesis, and neuro maturation compared with ND chips (Fig. 2d). Gene set enrichment analysis (GSEA) also showed that genes involved in fatty acid and bile acid metabolism are upregulated in healthy control and ND + SP chips compared to ND chips (Fig. 2e). Consistent with these findings, when we further analyzed cellular uptake of fatty acids using a fluorescently labeled dodecanoic acid, we found a 3.5-fold reduction in fatty acid uptake in ND chips compared to healthy chip controls, and treatment with SP significantly reversed this effect (Fig. 3a and b). Sideview imaging clearly showed that the fluorescent FAs were internalized by the epithelial cells which also produced mucus (Fig. 3c). Further, the fluorescent images with WGA and FA revealed that the apical surface of microvilli uptake FA, while the epithelial cells concurrently produce mucus (Supplementary Figure S3c). Consistent with these findings, SP treatment appeared to increase the expression levels of genes encoding proteins involved in fatty acid uptake (APOB, FABP1, FABP2, FASN) (Fig. 3d). Additionally, genes related to absorption of vital nutrients, such as iron (DMT1, FPN), exhibited lower expression in ND chips in comparison to healthy controls whereas SP treatment resulted in increased expression of these genes (Supplementary Figure S5a and b).

### SP suppresses nutritional deficiency-associated inflammatory responses

**Fig. 4:**
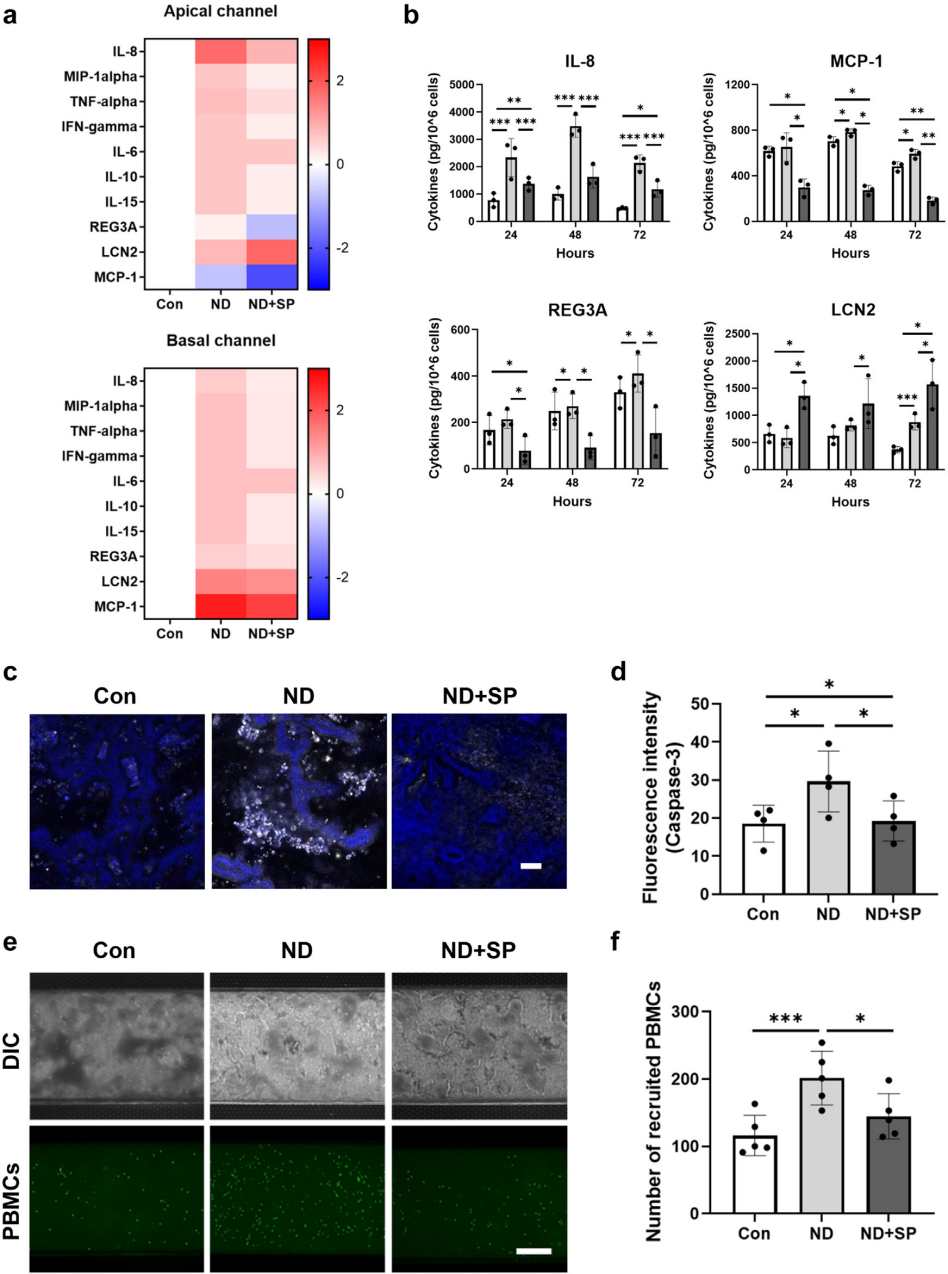
**Effect of SP on inflammatory responses associated with nutritional deficiency.** (a) Heatmap showing differential expression of 10 cytokines secreted into the apical (epithelium) and the basal (endothelium) channels of Intestine Chips at day 3 after SP treatment, measured by Luminex assay. The color-coded scale represents the log_2_ fold change in expression. (b) Production of four key cytokines on day 1, day 2, and day 3 after SP treatment. White (Con), gray (ND), and dark gray (ND + SP). ∗*p* < 0.05, ∗∗*p* < 0.01, ∗∗∗*p* < 0.001 by a one-way ANOVA. (c) Immunofluorescence imaging of the epithelial channel of Intestine Chips stained for Caspase-3 (white) and Hoechst 33342 (blue). Scale bar = 100 μm. (d) Differences in fluorescence intensity of Caspase-3 from Intestine Chip images (c). ∗*p* < 0.05, by a one-way ANOVA. (e) Recruitment of PBMCs in the Intestine Chip. PBMCs were stained with CellTracker Green™ CMFDA. Scale bar = 50 μm. (f) Quantification of recruited PBMCs in the top channel of chips from Intestine Chip images (e). ∗*p* < 0.05, ∗∗∗*p* < 0.001 by a one-way ANOVA.

Malnutrition-associated enteric function also commonly results in enhanced intestinal inflammation.^17^ Consistent with this clinical observation, we detected increased protein levels of multiple intestinal immunomodulators, including interleukin-8 (IL-8), MIP-1α, TNF-α, IFN-γ, IL-10, IL-15, REG3A, and lipocalin-2 (LCN2) in ND Intestine Chips compared to healthy control chips (Fig. 4a and b). Once again, treatment with SP suppressed this inflammatory response by reducing levels of most of these cytokines while further increasing the production of LCN2 (Fig. 4b), which has been reported to maintain intestinal microbiota homeostasis and protect against intestinal inflammation.^18^ This increase in inflammation observed in the ND chips was accompanied by epithelial damage and apoptosis, as indicated by increased Caspase-3 immunostaining, and again this was reversed by SP treatment (Fig. 4c and d). Furthermore, when we introduced human peripheral blood mononuclear cells (PBMCs) into the endothelium-lined vascular channel of the chip, increased numbers of these immune cells became adherent to the surface of the epithelium in ND chips compared with healthy chip controls. Importantly, this inflammatory recruitment of immune cells was also significantly inhibited by SP treatment (Fig. 4e and f). We also performed flow cytometric analysis of the adherent immune cells, and did not detect any significant changes in the distribution of T cells, B cells, or monocytes under the different experimental conditions (Supplementary Figure S6).

## Discussion

The high incidence of low birth weight newborns in women with malnutrition is a critical problem in low-resource nations and a major international issue because it is associated with increased infant mortality and developmental delays in children. The serendipitous finding that SP antimalarial therapy reduces the incidence of lower birth weight infants in pregnant women in sub-Saharan Africa has raised the possibility that this antibiotic combination might have direct effects on maternal physiology that are independent of its antimicrobial actions. In the present study, we investigated this directly by leveraging Organ Chip technology to engineer human Intestine Chips lined with young adult female patient-derived duodenal epithelial cells interfaced with endothelial cells and by mimicking ND using nutrient-reduced growth medium lacking niacinamide and tryptophan. Niacin deficiency during pregnancy is a particular matter of concern due to its potential adverse effects on maternal and fetal health.^19^ We have previously demonstrated that human Intestine Chips cultured under these ND conditions replicate the EED phenotype observed clinically.^5^^,^^20^ For example, inflammatory diseases such as inflammatory bowel disease (IBD) are associated with certain antimicrobial genes like DUOX2 and DUOXA2, and elevated DUOX2 levels in the intestine often indicate increased inflammation and mucosal dysbiosis.^21^^,^^22^ We observed that exposure to ND in healthy donors did not dramatically alter to the expression of these genes. This is interesting because it may indicate that short-term malnutrition might not be sufficient to induce significant changes in those genes compared to chronic conditions like those observed in EED and IBD patients. Based on our transcriptomic data, expression of the LGR5 marker indicative of highly proliferative intestinal stem cells is reduced when ND chips are treated with SP (i.e., ND + SP vs. ND). SP and its associated reduction of inflammation may therefore switch the intestine from a regenerative state towards differentiation and increased functionality, including enhanced nutrient absorption. In terms of goblet cells, we observed that SP enhanced expression level of MUC2, which is produced by goblet cells, when compared with ND group, suggesting another potential beneficial effect of SP on the intestinal epithelium.

Importantly, our results show that SP treatment significantly improved conditions of villus blunting, compromised barrier function, mucus layer thinning, and reduced nutrient absorption, and it also prevented epithelial cell death, which are all induced by ND conditions in human Intestine Chips. SP treatment also suppresses the release of inflammatory cytokines and inhibits the recruitment of circulating immune cells. Thus, SP does indeed have direct effects on the human adult female intestine that could in part explain the improved birth weights seen in malnourished mothers who were treated with SP as an antimalarial therapy.^4^^,^^23^

SP appears to alleviate intestinal dysfunction induced by nutritional deficiencies in multiple ways based on our in vitro studies with human Intestine Chips. First, it restores a more normal villus architecture with increased intestinal surface area that is available to absorb digested food molecules. Second, SP treatment of the ND intestine also improves the secretion of mucins and increases the thickness of the intestinal mucus layer that normally lubricates the epithelial surface and protects the epithelium from commensal microorganisms, invading pathogens, and other environmental irritants.^24^ Third, SP induces expression of multiple genes that are crucial for the efficient absorption of nutrients and fatty acids, which are extremely important during pregnancy for both maternal and fetal health.^25^ In particular, we found that ND conditions reduce fatty acid absorption in the intestinal epithelium and that treatment with SP counters this inhibition. Genes involved in fatty acid uptake, transport, and metabolism, which are important for maintaining both glucose and energy homeostasis,^26^ are also downregulated in the ND condition compared with healthy controls, and again SP treatment largely reverses this effect. This could be due in large part to the decreased villus surface area we observed. These findings also could potentially explain a past clinical finding that showed impaired fatty acid metabolism in children afflicted by EED.^27^ However, our data suggest that nutritional deficiency can lead to diminished fatty acid uptake, even in a healthy intestinal environment.

Interestingly, we found that SP treatment stimulates the metabolism of bile acids, which are known to play multiple roles in lipid metabolism and serve as signaling molecules in the small intestine by modulating lipid, glucose, and energy homeostasis, in addition to supporting the absorption of dietary lipids. Alterations in bile acid metabolism are known to occur during pregnancy and deficiency in essential nutrients such as vitamin, iron, and minerals can impact not only nucleoside transporters, but also the production and regulation of neurotransmitters. Hence, it is also possible that SP might have a positive effect on metabolism during pregnancy, although this would need to be demonstrated experimentally. However, it would be extremely difficult to obtain intestinal biopsies from pregnant women for ethical reasons. While it is possible that female hormones might further alter these effects, our results clearly demonstrate that the SP antibiotic combination can directly affect intestinal structure and function, as well as multiple biological process and pathways that play a pivotal role in maintaining homeostasis, nutrient absorption, and metabolism within the human body.

In the living intestine, there is an intricate interplay between intestinal epithelial cells and the mucosal immune system,^28^ which becomes deregulated leading to increased inflammation in patients with EED or other forms of intestinal dysfunction. Similarly, we found that culturing healthy Intestine Chips lined by epithelium from young adult female donors in the presence of ND medium resulted in increased production of multiple pro-inflammatory cytokines, which is consistent with what we observed previously in chips lined by cells from EED patients when exposed to nutritional deficiency.^5^ Nutritional deficiency, whether due to a lack of essential vitamins, minerals, or macronutrients, can lead to a state of subclinical inflammation. This inflammation is characterized by the release of pro-inflammatory cytokines, TNF-α and IL-8, which serve as key mediators in the immune response.^29^, ^30^, ^31^ Notably, these elevated cytokine levels in the ND Intestine Chips reduced significantly when the chips were treated with SP. The only exception was LCN2, which was induced by SP. This is interesting because LCN2 plays a role in innate immunity and serves as a host defense mechanism against infection by sequestering iron-containing compounds called siderophores, which are essential for bacterial growth. In addition to its iron-sequestering function, LCN2 can modulate immune responses, for example, by activating certain pro-inflammatory pathways (e.g., NF-κB).^32^ Consistent with these observations, we found that when we introduced PBMCs into the endothelium-lined vascular channel of the Intestine Chips, ND increased immune cell recruitment whereas SP treatment greatly reduced this response.

Our results show that SP treatment can modulate functions of the female intestine in vitro, including nutrient absorption and inflammation, that are critical for the health of the mother and the developing fetus. However, we did not explicitly model the pregnancy state in these studies and hence, future clinical studies will be necessary to test this prediction directly in malnourished pregnant women. In patients, SP treatment also will likely need to be supplemented with key nutrients, such as vitamins and minerals, that can help to further counteract the pro-inflammatory effects of nutritional deficiencies. These interventions hold potential not only in preventing chronic diseases but also in managing existing inflammatory conditions.^33^ We focused on the duodenum in this study because it is a critical site for the absorption of essential nutrients and drugs particularly during pregnancy, when nutrient absorption, drug metabolism, and overall gut health are of heightened importance due to the increased nutrient demands for sustenance of both maternal and fetal well-being.^34^ Although we did not specifically address differences in intestinal function related to pregnancy, the model we employed does mimic the effects of malnutrition and a similar approach can be used to explore how ileum, jejunum, and colon respond to SP and contribute to maternal health in the future, as we have previously described Organ Chip models for each of these intestinal regions.^12^^,^^35^

Taken together, these results show that SP can indeed have multiple direct effects on human intestinal structure and function that could contribute to the serendipitous observation of reduced incidence of low birth weight newborns, which is seen in pregnant women after anti-malaria treatment with SP. Hopefully, these results will stimulate future in-depth clinical studies to test this prediction directly as our findings suggest that SP should be further explored as a potential treatment for this important global health issue. The human Intestine Chip also may prove useful in addressing other questions related to intestinal physiology and pathophysiology that are of interest to the global health community.

## Contributors

S.K. led this study, and S.K., G.G., and D.E.I. designed the overall research. S.K., A.N., and P.P. performed experiments. S.K., V.H., A.J., G.G., and D.E.I. analyzed and interpreted all the data. D.T.B. established human intestinal organoids. S.K., G.G., and D.E.I. verified the underlying data. S.K. and D.E.I. wrote the article with input from other authors. All authors reviewed and approved the final version of the manuscript.

## Data sharing statement

The datasets and analysis will be available upon request. All requests should be addressed to the corresponding author (don.ingber@wyss.harvard.edu).

## Declaration of interests

D.E.I. holds equity in Emulate, Inc., chairs its scientific advisory board, and is a member of its board of directors. The other authors declare no competing interests.

## References

[1] Gernand A.D., Schulze K.J., Stewart C.P., West K.P., Christian P. (2016). Micronutrient deficiencies in pregnancy worldwide: health effects and prevention. Nat Rev Endocrinol.

[2] Abu-Saad K., Fraser D. (2010). Maternal nutrition and birth outcomes. Epidemiol Rev.

[3] Deloron P., Bertin G., Briand V., Massougbodji A., Cot M. (2010). Sulfadoxine/pyrimethamine intermittent preventive treatment for malaria during pregnancy. Emerg Infect Dis.

[4] Kayentao K., Garner P., van Eijk A.M. (2013). Intermittent preventive therapy for malaria during pregnancy using 2 vs 3 or more doses of sulfadoxine-pyrimethamine and risk of low birth weight in Africa: systematic review and meta-analysis. JAMA.

[5] Bein A., Fadel C.W., Swenor B. (2022). Nutritional deficiency in an intestine-on-a-chip recapitulates injury hallmarks associated with environmental enteric dysfunction. Nat Biomed Eng.

[6] Soderholm A.T., Pedicord V.A. (2019). Intestinal epithelial cells: at the interface of the microbiota and mucosal immunity. Immunology.

[7] Zheng D., Liwinski T., Elinav E. (2020). Interaction between microbiota and immunity in health and disease. Cell Res.

[8] Kasendra M., Tovaglieri A., Sontheimer-Phelps A. (2018). Development of a primary human small intestine-on-a-chip using biopsy-derived organoids. Sci Rep.

[9] Sato T., Stange D.E., Ferrante M. (2011). Long-term expansion of epithelial organoids from human colon, adenoma, adenocarcinoma, and Barrett's epithelium. Gastroenterology.

[10] Elfer K.N., Sholl A.B., Wang M. (2016). DRAQ5 and Eosin ('D&E') as an analog to hematoxylin and eosin for rapid fluorescence histology of fresh tissues. PLoS One.

[11] Bein A., Kim S., Goyal G. (2021). Enteric coronavirus infection and treatment modeled with an immunocompetent human intestine-on-a-chip. Front Pharmacol.

[12] Sontheimer-Phelps A., Chou D.B., Tovaglieri A. (2020). Human colon-on-a-chip enables continuous in vitro analysis of colon mucus layer accumulation and physiology. Cell Mol Gastroenterol Hepatol.

[13] Kim H.J., Ingber D.E. (2013). Gut-on-a-chip microenvironment induces human intestinal cells to undergo villus differentiation. Integr Biol.

[14] Subramanian A., Tamayo P., Mootha V.K. (2005). Gene set enrichment analysis: a knowledge-based approach for interpreting genome-wide expression profiles. Proc Natl Acad Sci U S A.

[15] Morrison J.L., Regnault T.R. (2016). Nutrition in pregnancy: optimising maternal diet and fetal adaptations to altered nutrient supply. Nutrients.

[16] Vrijkotte T.G., Algera S.J., Brouwer I.A., van Eijsden M., Twickler M.B. (2011). Maternal triglyceride levels during early pregnancy are associated with birth weight and postnatal growth. J Pediatr.

[17] Guerrant R.L., Oria R.B., Moore S.R., Oria M.O., Lima A.A. (2008). Malnutrition as an enteric infectious disease with long-term effects on child development. Nutr Rev.

[18] Moschen A.R., Gerner R.R., Wang J. (2016). Lipocalin 2 protects from inflammation and tumorigenesis associated with gut microbiota alterations. Cell Host Microbe.

[19] Palawaththa S., Islam R.M., Illic D. (2022). Effect of maternal dietary niacin intake on congenital anomalies: a systematic review and meta-analysis. Eur J Nutr.

[20] Nyaradi A., Li J., Hickling S., Foster J., Oddy W.H. (2013). The role of nutrition in children's neurocognitive development, from pregnancy through childhood. Front Hum Neurosci.

[21] Kummerlowe C., Mwakamui S., Hughes T.K. (2022). Single-cell profiling of environmental enteropathy reveals signatures of epithelial remodeling and immune activation. Sci Transl Med.

[22] Grasberger H., Gao J., Nagao-Kitamoto H. (2015). Increased expression of DUOX2 is an epithelial response to mucosal dysbiosis required for immune homeostasis in mouse intestine. Gastroenterology.

[23] ter Kuile F.O., van Eijk A.M., Filler S.J. (2007). Effect of sulfadoxine-pyrimethamine resistance on the efficacy of intermittent preventive therapy for malaria control during pregnancy: a systematic review. JAMA.

[24] Grondin J.A., Kwon Y.H., Far P.M., Haq S., Khan W.I. (2020). Mucins in intestinal mucosal defense and inflammation: learning from clinical and experimental studies. Front Immunol.

[25] Duttaroy A.K., Basak S. (2020). Maternal dietary fatty acids and their roles in human placental development. Prostaglandins Leukot Essent Fatty Acids.

[26] Rui L. (2014). Energy metabolism in the liver. Compr Physiol.

[27] Ramakrishnan G., Petri W.A. (2017). Secondary carnitine deficiency in environmental enteric dysfunction. EBioMedicine.

[28] Takiishi T., Fenero C.I.M., Camara N.O.S. (2017). Intestinal barrier and gut microbiota: shaping our immune responses throughout life. Tissue Barriers.

[29] Takele Y., Adem E., Getahun M. (2016). Malnutrition in healthy individuals results in increased mixed cytokine profiles, altered neutrophil subsets and function. PLoS One.

[30] Fatyga P., Pac A., Fedyk-Lukasik M., Grodzicki T., Skalska A. (2020). The relationship between malnutrition risk and inflammatory biomarkers in outpatient geriatric population. Eur Geriatr Med.

[31] Kim M.H., Kim A., Yu J.H., Lim J.W., Kim H. (2014). Glutamine deprivation induces interleukin-8 expression in ataxia telangiectasia fibroblasts. Inflamm Res.

[32] Kim S.L., Shin M.W., Kim S.W. (2022). Lipocalin 2 activates the NLRP3 inflammasome via LPS-induced NF-kappaB signaling and plays a role as a pro-inflammatory regulator in murine macrophages. Mol Med Rep.

[33] Iddir M., Brito A., Dingeo G. (2020). Strengthening the immune system and reducing inflammation and oxidative stress through diet and nutrition: considerations during the COVID-19 crisis. Nutrients.

[34] Meyer A.M., Caton J.S. (2016). Role of the small intestine in developmental programming: impact of maternal nutrition on the dam and offspring. Adv Nutr.

[35] Gazzaniga F.S., Camacho D.M., Wu M. (2021). Harnessing colon chip technology to identify commensal bacteria that promote host tolerance to infection. Front Cell Infect Microbiol.

