# Supplementary material for: Direct therapeutic effect of sulfadoxine-pyrimethamine on nutritional deficiency-induced enteric dysfunction in a human Intestine Chip
Source: eBioMedicine. 2023 Dec 14;99:104921. doi: 10.1016/j.ebiom.2023.104921 (PMC10733102; doi:10.1016/j.ebiom.2023.104921)
Supplement: Supplementary Figures S1–S6 [file mmc1.docx]

**Direct therapeutic effect of sulfadoxine-pyrimethamine on nutritional deficiency-induced enteric dysfunction in a human intestine chip**

Seongmin Kim^1^, Arash Naziripour^1^, Pranav Prabhala^1^, Viktor Horváth^1^, Abidemi Junaid^1^, David T. Breault^2,3,4^, Girija Goyal^1^, Donald E. Ingber^1,5,6^*

**SUPPLEMENTARY DATA**


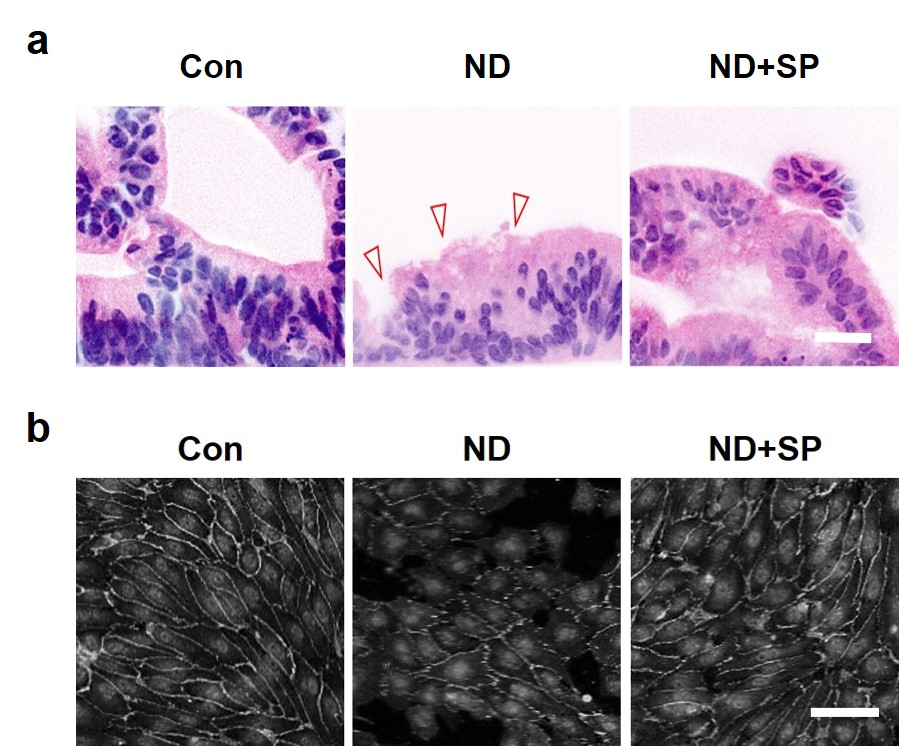


***Supplementary Fig. 1*: Morphological characteristics of Intestine Chips.** (a) Pseudo-H&E stained images of the intestinal epithelium on Intestine Chips. Red arrows indicate regions of potentially damaged epithelium and pseudostratified cytoskeleton. The chips were sectioned using a vibratome into 40-60 µm, and the stained specimens were placed on thin cover glass (0.13-0.16 mm thick) for imaging. Scale bar = 50 µm. (b) Immunofluorescence imaging of the basal channel (endothelium) of Intestine Chips stained with VE-Cadherin (white) and Hoechst (gray). Scale bar = 50 µm.


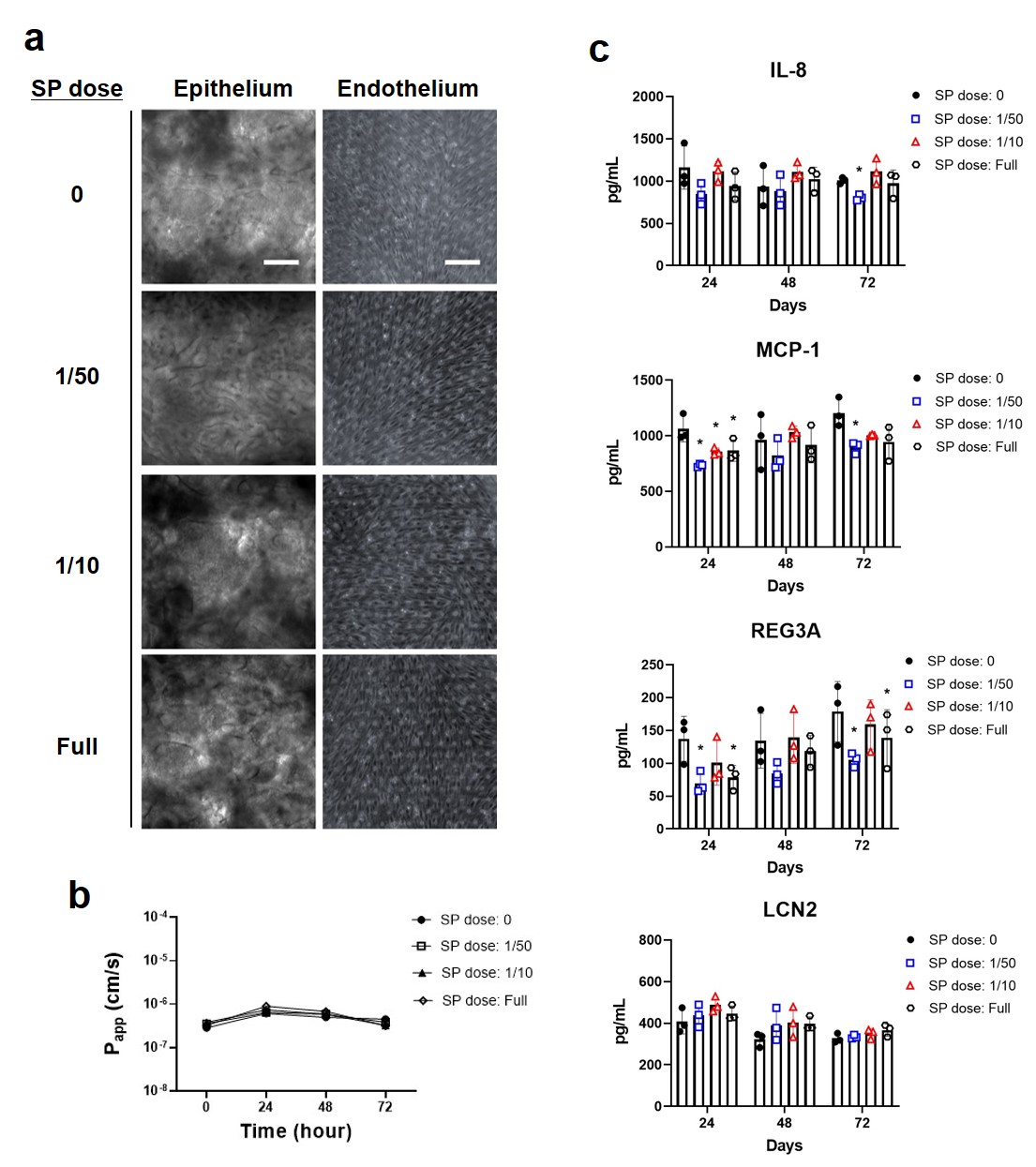


***Supplementary Fig. 2*: Optimal dose-finding for an Intestine Chip model.** (a) Phase-contrast imaging of Intestine Chips. Intestine Chips were treated with DMSO only (control) or different doses of SP dissolved in DMSO. The epithelial channel of the Intestine Chip was perfused with the calculated acute duodenal dose ([133 µg S + 6·7 µg P]/mL) of this formulation as well as lower doses (1/50th [2·66 µg S + 0·13 µg P]/mL and 1/10th [13·3 S + 0·67 µg P]/mL) in culture medium. Scale bar = 100 µm. (b) Apparent permeability (*P_app_*) after SP treatment with different doses. (c) Cytokine analysis at day 1, day 2, and day 3 after SP treatment was measured by Luminex assay in the effluent from the apical (epithelium) channel of Intestine Chips. **p* < 0·05 by a one-way ANOVA.


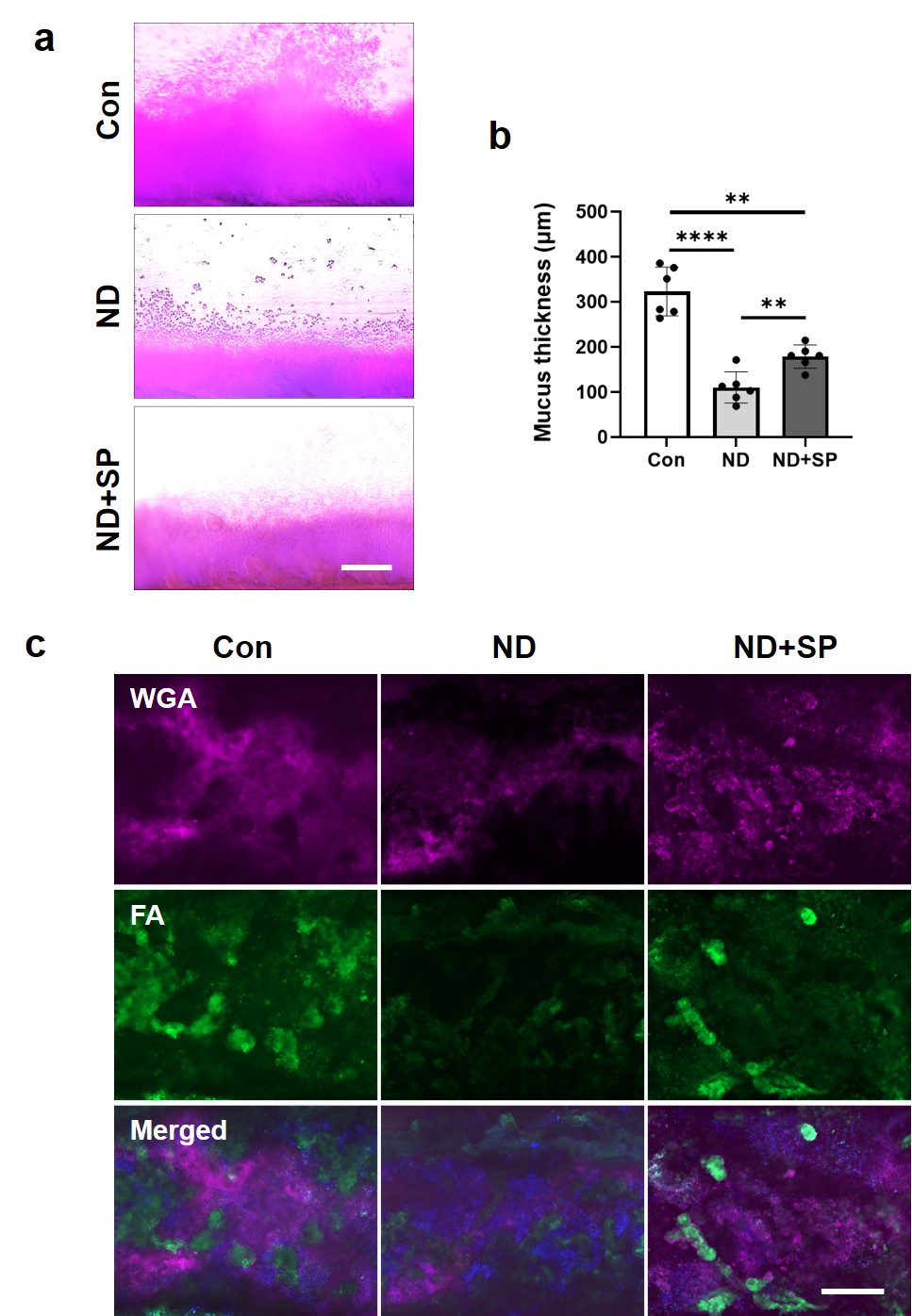


***Supplementary Fig. 3*: Characterization of mucus production and fatty acid uptake.** (a) Fluorescent microscopic views of vertical cross-sections of Intestine Chips showing the mucus layer stained positively with fluorescent WGA lectin (magenta) overlying the darkened intestinal epithelium at the base of this view. Scale bar = 200 µm. (b) Graph showing differences in mucus thickness in control, ND, and ND+SP chips. ***p* < 0·01, *****p* < 0·0001 by a one-way ANOVA. (c) Top-down views fluorescent imaging of Intestine Chips showing mucus secretion and FA uptake in the apical surface of epithelium. Wheat germ agglutinin (WGA)-Alexa 633 (ThermoFisher), fluorescently-labeled (Alexa 488) dodecanoic fatty acid, and Hoechst 33342 were used for fluorescent microscopic imaging. Scale bar = 100 µm.


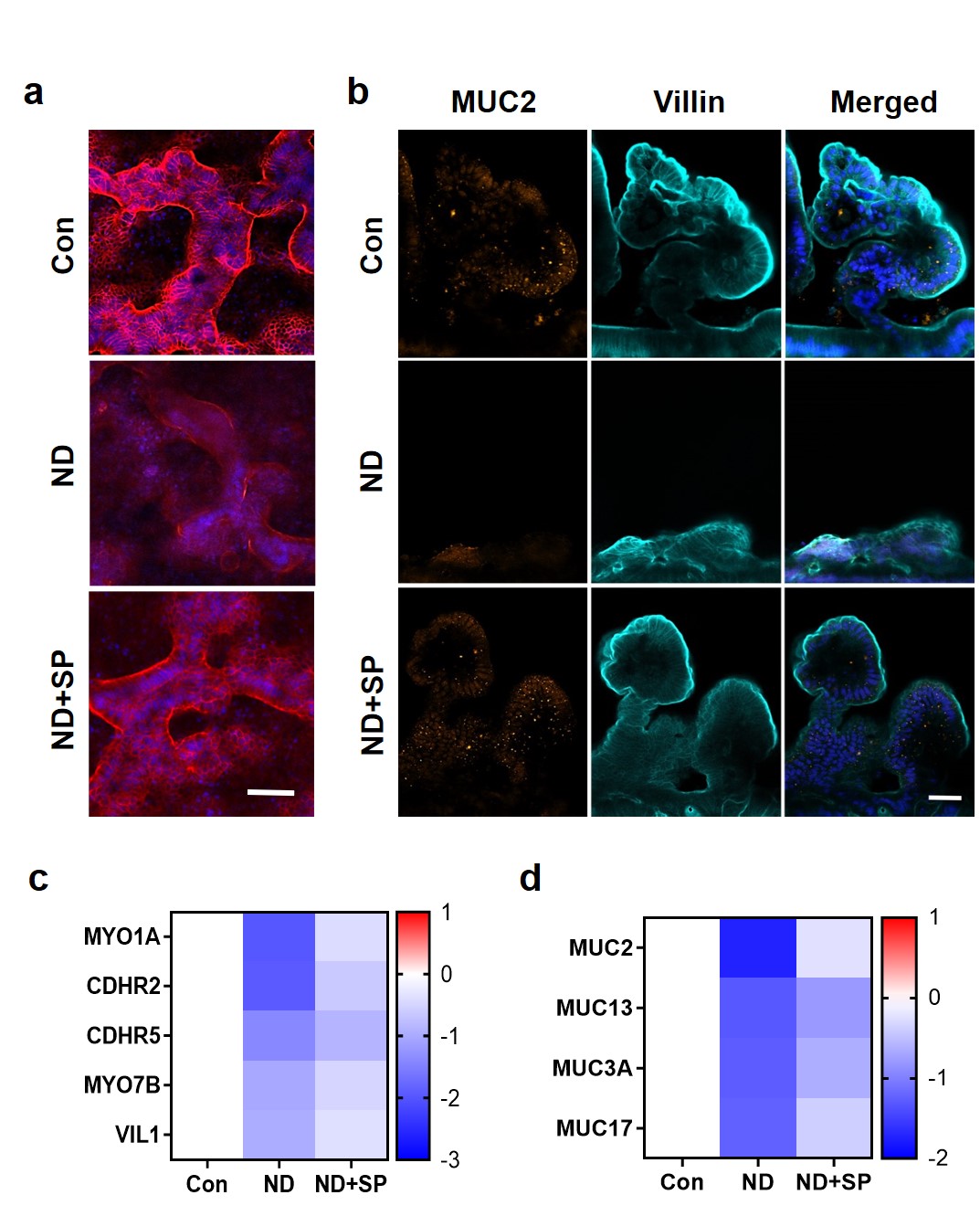


***Supplementary Fig. 4*: Altered intestinal villus atrophy and transcriptomic profiles.** (a) Top-down views fluorescent imaging of Intestine Chips showing the F-actin in the epithelium. Scale bar = 50 µm. (b) Immunofluorescence imaging of cross-sectioned Intestine Chips showing microvilli and mucin, orange: MUC2, cyan: Villin, blue: Hoechst 33342. Scale bar = 50 µm. (c) Heatmap showing differential expression of key genes associated with microvilli of the epithelial cells in the small intestine. The color-coded scale represents the log_2_ fold change in expression. (d) Heatmap showing differential expression of key genes associated with mucin in the small intestine. The color-coded scale represents the log_2_ fold change in expression.


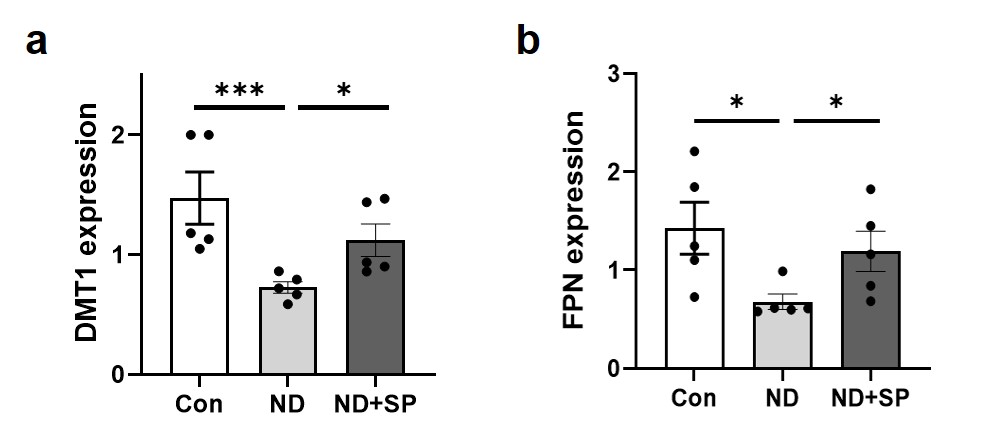


***Supplementary Fig. 5*: Intestinal expression of genes involved in iron absorption.** Graphs showing the fold change in the expression levels of human (a) DMT1 and (b) FPN. The TaqMan qPCR assay was carried out. **p* < 0·05, ****p* < 0·001 by a one-way ANOVA.


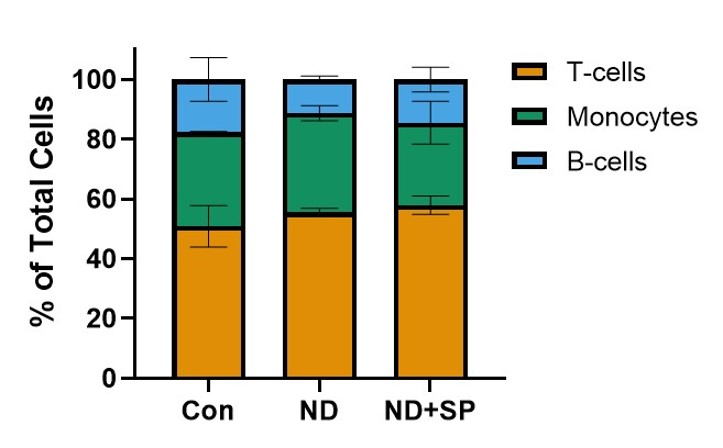


***Supplementary Fig. 6*: Composition of recruited immune cells.** PBMCs were collected from Intestine Chips and identified by FACS. Orange (T-cells by CD3), green (Monocytes by CD14), and blue (B-cells by CD19).
